# Performance of Two HCV RNA Assays during Protease Inhibitor-Based Triple Therapy in Patients with Advanced Liver Fibrosis and Cirrhosis

**DOI:** 10.1371/journal.pone.0110857

**Published:** 2014-11-12

**Authors:** Benjamin Maasoumy, Bela Hunyady, Vincenza Calvaruso, Mihály Makara, Johannes Vermehren, Attila Haragh, Simone Susser, Birgit Bremer, Gavin Cloherty, Michael P. Manns, Antonio Craxì, Heiner Wedemeyer, Christoph Sarrazin

**Affiliations:** 1 Department of Gastroenterology, Hepatology and Endocrinology, Hannover Medical School, Hanover, Germany; 2 Somogy County Kaposi Mor Teaching Hospital, Kaposvar, Hungary; 3 University of Pecs, Pecs, Hungary; 4 Section of Gastroenterology, Di.Bi.M.I.S., University of Palermo, Palermo, Italy; 5 Outpatient Clinic, Saint Laszlo Hospital, Budapest, Hungary; 6 Department of Medicine 1, JW Goethe-University Hospital, Frankfurt am Main, Germany; 7 Abbott Molecular, Des Plaines, Illinois, United States of America; University of Alberta, Canada

## Abstract

**Introduction:**

On-treatment HCV RNA measurements are crucial for the prediction of a sustained virological response (SVR) and to determine treatment futility during protease inhibitor-based triple therapies. In patients with advanced liver disease an accurate risk/benefit calculation based on reliable HCV RNA results can reduce the number of adverse events. However, the different available HCV RNA assays vary in their diagnostic performance.

**Aim:**

To investigate the clinical relevance of concordant and discordant results of two HCV RNA assays during triple therapy with boceprevir and telaprevir in patients with advanced liver fibrosis/cirrhosis.

**Methods:**

We collected on-treatment samples of 191 patients with advanced liver fibrosis/cirrhosis treated at four European centers for testing with the Abbott RealTime (ART) and COBAS AmpliPrep/COBAS TaqMan HCV v2.0 (CTM) assays.

**Results:**

Discordant test results for HCV RNA detectability were observed in 23% at week 4, 17% at week 8/12 and 9% at week 24 on-treatment. The ART detected HCV RNA in 41% of week 4 samples tested negative by the CTM. However, the positive predictive value of an undetectable week 4 result for SVR was similar for both assays (80% and 82%). Discordance was also found for application of stopping rules. In 27% of patients who met stopping rules by CTM the ART measured levels below the respective cut-offs of 100 and 1000 IU/ml, respectively, which would have resulted in treatment continuation. In contrast, in nine patients with negative HCV RNA by CTM at week 24 treatment would have been discontinued due to detectable residual HCV RNA by the ART assay. Importantly, only 4 of these patients failed to achieve SVR.

**Conclusion:**

Application of stopping rules determined in approval studies by one assay to other HCV RNA assays in clinical practice may lead to over and undertreatment in a significant number of patients undergoing protease inhibitor-based triple therapy.

## Introduction

Chronic hepatitis C is a major cause for liver transplantation and hepatocellular carcinoma worldwide [1], [2]. A successful viral eradication leads to a significant improvement of the overall survival rate and reduces liver related morbidity [3]. Efficacy of antiviral therapy markedly increased with the development of direct acting antiviral agents (DAAs). In 2011, the first generation of DAAs, the protease inhibitors (PIs) telaprevir (TVR) and boceprevir (BOC), were approved for treatment of chronic hepatitis C virus (HCV) genotype (GT) 1 infection [4]–[8]. More recently, a third PI, simeprevir (SMV) has been approved in the US, Canada and Japan [9]. Furthermore, the polymerase-inhibitor sofosbuvir (SOF) was most recently marketed in the US and some European countries. More DAAs will soon be approved. Second generation DAAs will certainly lead to an improved safety and efficacy of HCV treatment [9]–[11]. However, so far standard treatment in most GT1 patients is still based on pegylated-interferon and ribavirin (P/R). Furthermore, due to high costs, it will likely take quite some time until SOF and SMV will be approved and available in most parts of the world, as several countries have only recently attained access to BOC and TVR or are even still awaiting the approval or reimbursement of first generation PIs.

In several real-life cohorts of patients with advanced liver disease the frequency of serious adverse events was high when treated with first generation PI-based triple therapy. In particular severe infections and hepatic decompensations were a significant problem. Even lethal complications have been documented. Furthermore, efficacy was also lower compared with those in patients with no or only mild fibrosis [12]–[14]. Thus, in order to ensure a reasonable risk/benefit ratio in patients with urgent need of antiviral therapy, but increased risks of serious adverse events, it is crucial to establish predictive factors for a sustained virological response. While there are a number of baseline predictors, the most important response parameter during treatment remains HCV RNA viral kinetics [15]. Here in particular, the difference between undetectable HCV RNA and residual HCV viremia may be of high prognostic value [16], [17]. In addition, quantitative HCV RNA levels at certain time points during PI-based triple therapy determine treatment futility [2]. By early discontinuation of unlikely to succeed therapies, accurate futility rules may prevent not only unnecessary side-effects but also reduce therapy-related costs.

A number of different HCV RNA assays with variable sensitivities and accuracies are used in clinical practice. However, only little is known regarding the extent to which different assay performances may influence the management of PI-based triple therapies including determination of treatment duration and early discontinuation of antiviral therapy. Furthermore, it is not clear whether individual assay performances may lead to differences in the predictive value and/or the sensitivity to identify patients who are at risk of treatment failure and for whom the risk of treatment associated toxicity might be unacceptable.

We here compared the performance of two HCV RNA assays, the Abbott RealTime HCV Test (ART) and the COBAS AmpliPrep/COBAS TaqMan HCV Test v2.0 (CTM) [18], [19], in patients with advanced liver fibrosis/cirrhosis who were treated with TVR- and BOC-based triple therapy in four European centers. We analyzed the impact of the two assays on stopping rules and the predictive value for achieving SVR.

## Patients and Methods

### Patients

A total number of 191 HCV genotype 1 monoinfected patients was included from four European study sites: Hannover Medical School (Hanover, Germany), University of Palermo (Palermo, Italy), Saint László Hospital (Budapest, Hungary) and Somogy County Kaposi Mór Teaching Hospital (Kaposvár, Hungary). Patients with HBV or HIV infection were excluded. HCV subgenotype was available for 169 (88%) patients, of whom 87% were infected with HCV GT 1b. All patients had advanced liver fibrosis or cirrhosis (METAVIR F3/F4) as determined by liver biopsy, transient elastography or obvious clinical signs. Patients were treated with TVR (n = 65) or BOC (n = 126) in combination with P/R according to the respective prescribing information and international guidelines [20], [21].

### HCV RNA measurements

Patient samples were collected at 4, 8 (BOC), 12 (TVR) and/or 24 weeks after the start of PI treatment, the key decision time points for response-guided treatment and/or stopping criteria [2], [20], [21]. All samples were first tested with the COBAS AmpliPrep/COBAS TaqMan HCV Test v2.0 (CTM) (limit of quantification  =  LOQ: 15 IU/ml; limit of detection  =  LOD: 15 IU/ml) and retrospectively re-tested with the Abbott RealTime HCV Test (ART) (LOQ and LOD: 12 IU/ml) according to the manufactures' instructions. Treatment decisions were based on the CTM results. At each time point, only those patients in whom the respective sample volume was sufficient for re-testing with the ART were included in the later analysis.

### Selection of samples for the analysis of the concordance in patients with no or only residual viremia

For the analysis of concordance in differentiating samples with low residual viremia from those that were HCV RNA negative, only samples with an HCV RNA <50 IU/ml in at least one of the two tests were considered.

### Selection of samples for determining the assays' concordance at HCV RNA levels close to the threshold of stopping rules

One objective of this study was to determine the concordance between the two assays at HCV RNA levels leading to discontinuation of therapy due to the recommended stopping criteria. For this purpose only those samples were considered that had an HCV RNA level of ≥50 IU/ml in at least one of the two assays. At week 24 all samples with detectable HCV RNA in at least one of the two tests were considered.

### Statistics

Data were collected with Microsoft Excel (Microsoft, Redmond, Washington, USA) and analyzed with GraphPad Prism for Mac (version 6.0; GraphPad Software Inc., La Jolla, California, USA).

### Ethics

This study was performed according to the Declaration of Helsinki. The local ethical committee of Hannover Medical School approved the retrospective, anonymous retesting of patient samples, and the anonymous analyzing of patient data without the need for a written informed consent.

## Results

### Assay concordance in low viremic or HCV RNA negative samples obtained during triple therapy

Concordance between the two assays in classifying samples as HCV RNA positive or negative varied among the different time points. Overall, we observed an increasing concordance during later stages of therapy starting at 77% at week 4, 83% at week 8/12 and 91% at week 24 (Table 1).

**Table 1:** Concordance and discordance between the ART and the CTM in classifying samples as HCV RNA undetectable or low viremic (<50 IU/ml) obtained 4, 8 (BOC), 12 (TVR) and 24 weeks after the start of PI-based treatment.

			CTM			
			Not detectable	Detectable	Discordance	Overall Concordance/Discordance
4 weeks after PI therapy	ART	Not Detectable	17	4	19% (4/21)	77% (54/70)/23% (16/70)
		Detectable	12	37		
		Discordance	41% (12/29)			
4 weeks after TVR therapy	ART	Not Detectable	15	4	21% (4/19)	73% (41/56)/27% (15/56)
		Detectable	11	26		
		Discordance	42% (11/26)			
4 weeks after BOC therapy	ART	Not Detectable	2	0	0% (0/2)	93% (13/14)/7% (1/14)
		Detectable	1	11		
		Discordance	33% (1/3)			
8/12 weeks after PI therapy	ART	Not Detectable	78	8	9% (8/86)	83% (104/126)/17% (22/126)
		Detectable	14	26		
		Discordance	15% (14/92)			
12 weeks after TVR therapy	ART	Not Detectable	45	2	4% (2/47)	87% (46/53)/13% (7/53)
		Detectable	5	1		
		Discordance	10% (5/50)			
8 weeks after BOC therapy	ART	Not Detectable	33	6	15% (6/39)	80% (58/73)/20% (15/73)
		Detectable	9	25		
		Discordance	21% (9/42)			
24 weeks after PI therapy	ART	Not Detectable	93	1	1% (1/94)	91% (99/109)/9% (10/109)
		Detectable	9	6		
		Discordance	9% (9/102)			
24 weeks after TVR therapy	ART	Not Detectable	38	0	0% (0/38)	93% (39/42)/7% (3/42)
		Detectable	3	1		
		Discordance	7% (3/41)			
24 weeks after BOC therapy	ART	Not Detectable	55	1	2% (1/56)	90% (60/67)/10% (7/67)
		Detectable	6	5		
		Discordance	10% (6/61)			

However, in week 4 samples the relative sensitivity to detect residual HCV RNA by the CTM assay was low. Retesting of these week 4 samples with the ART assay revealed detectable HCV RNA in 41%. In contrast, the CTM detected HCV RNA in 19% of the samples with an undetectable result in the ART assay (Table 1).

Similar results were observed at week 8/12 of therapy. Retesting with the ART detected HCV RNA in 14 samples that were not identified as HCV RNA positive in the initial measurement with the CTM. In contrast, the CTM also produced 8 positive results in samples classified as negative if retested with the ART (Table 1). However, due to a higher total number of negative tested samples reliability of an undetectable result was far higher compared with week 4 (ART: 93% vs. 81%; CTM: 85% vs. 59%). The observed differences between the two HCV RNA assays overall were not influenced by the used PI (TVR or BOC). Still, due to the shorter PI treatment duration, there were more positive samples in patients treated with BOC. Subsequently, reliability of an undetectable result was lower after 8 weeks of BOC treatment compared to samples obtained after 12 weeks of TVR treatment (Table 1).

At week 24, the vast majority of samples tested negative for HCV RNA by both assays (85%). Again a few samples (n = 9) tested negative by the CTM revealed detectable HCV RNA after retesting with the ART. In contrast, only a single sample that was not detected with the ART produced a positive result in the initial measurement with the CTM. Results for week 24 were also similar for both PIs (Table 1).

### Predictive value of concordant/discordant low viremic (<50 IU/ml) or negative HCV RNA results for the final treatment outcome

**Figure 1:**
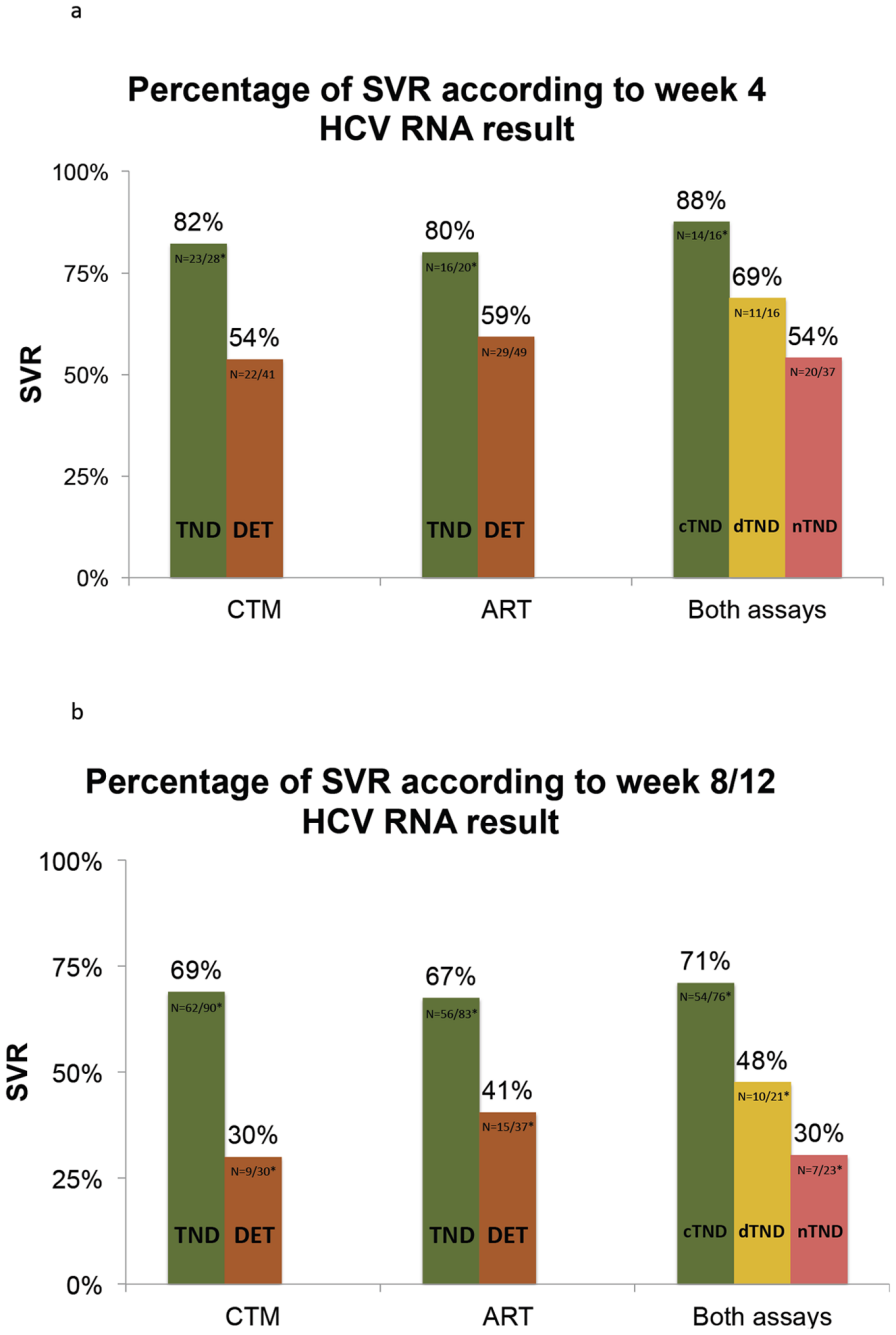
Predictive value of HCV RNA results in the CTM and ART at week 4 (a) and week 8/12 (b) after start of therapy with a protease inhibitor. TND  =  target not detected; DET  =  HCV RNA detected; TND/TND  =  HCV not detected in both assays; TND/DET  =  HCV RNA detected by only one assay; DET/DET: HCV RNA detected by both assays. *Patients with unavailable virological treatment outcome were excluded from analysis.

On-treatment HCV RNA results had a high prognostic value for the final treatment outcome. Patients with an undetectable HCV RNA result by CTM four weeks after PI therapy achieved a SVR in 82% (n = 23/28) compared to only 54% (n = 22/41) of those with detectable low viremia (HCV RNA <50 IU/ml). Similar results were documented for the ART with an 80% (n = 16/20) and a 59% (n = 29/49) SVR rate in those with an undetectable and a detectable low viremic week 4 HCV RNA result, respectively. Interestingly, SVR rate increased from 69% (n = 11/16) in patients with only one undetectable HCV RNA result up to 88% (n = 14/16) in those who were undetectable with both assays at week 4 (Figure 1a). Sensitivity to detect a patient with a later treatment failure by a low viremic week 4 sample was 83% for the ART, 79% for the CTM and 92% using both assays.

Similar results were observed at week 8/12. Patients with undetectable HCV RNA by CTM and ART at this stage were cured in 69% (n = 62/90) and 67% (n = 56/83), respectively. In contrast, among those with a detectable low viremic result by CTM, only 30% (n = 9/30) achieved SVR whereas still 41% (n = 15/37) were detectable with the ART. Nevertheless SVR chances were still markedly lower in those patients, in whom only one of the two assays produced a negative HCV RNA result compared to those, in whom testing with the CTM as well as retesting with the ART did not detect any HCV RNA (48% vs. 71%) (Figure 1b). Interestingly, not a single patient with quantifiable HCV RNA at week 8/12 (n = 8) in at least one of the two assays achieved SVR. Overall, data were similar for both PIs. However, all three patients that had still a detectable low viremic HCV RNA level after 12 weeks of TVR treatment by the CTM experienced a treatment failure, while SVR rate was 50% amongst the six patients that yielded a detectable level by ART. Sensitivity to identify a patient with a later treatment failure by residual viremia was 45% using the ART, 43% with the CTM and increased to 55% using both assays.

### Assay concordance in samples with quantifiable HCV RNA and in determining treatment futility

**Figure 2:**
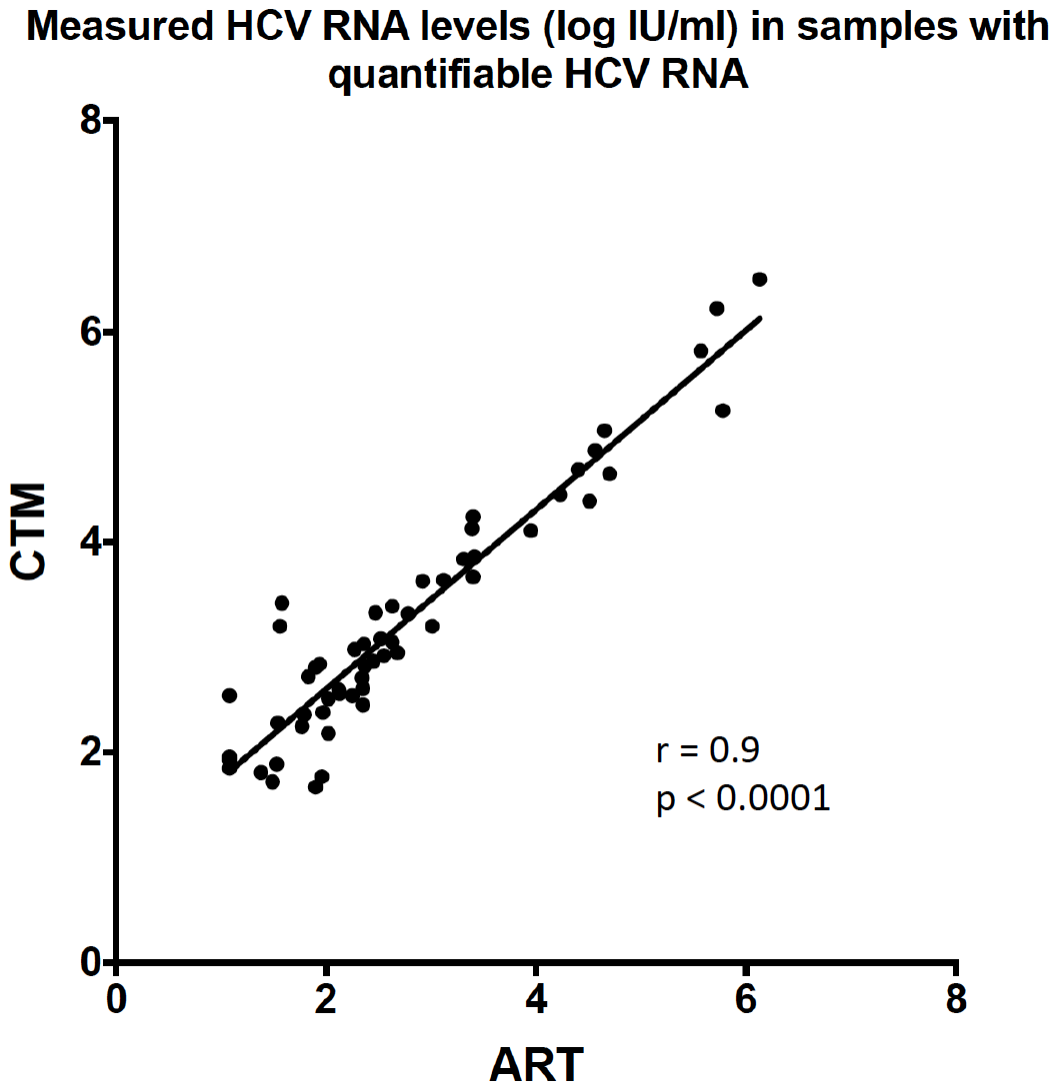
Correlation between the CTM and the ART in samples with quantifiable HCV RNA levels. r: Spearman correlation; p: p-value.

There were 56 samples available with quantifiable HCV RNA ≥50 IU/ml (range: 1.08–6.50 log IU/ml). Again, all samples were first tested with the CTM and afterwards retested with the ART. Correlation between the CTM and the ART was good in these samples (Spearman correlation: r = 0.9) (Figure 2). However, the CTM tended to yield higher levels compared with the ART. Overall, HCV RNA levels were higher with the CTM test in 91% of the samples. The mean log difference between the measured HCV RNA levels in both assays was 0.53 log IU/ml (range: 0.04–1.84 log IU/ml).

There were eight patients with an HCV RNA level ≥50 IU/ml after 4 weeks of TVR treatment (13% of all TVR treated patients). Two patients had a level ≥1000 IU/ml in the CTM assay, where stopping of all antiviral treatment is recommended. Interestingly, none of these patients had an HCV RNA level>1000 IU/ml in the ART and would therefore had been kept on treatment according to the ART assay. One of these patients continued treatment but experienced a virological breakthrough by week 12 of therapy. Similar results were observed in patients after four weeks of BOC treatment. Overall four patients had an HCV RNA result ≥50 IU/ml including two with a level>1000 IU/ml. All four samples had HCV RNA levels <50 IU/ml if re-tested with the ART (Table 2).

**Table 2:** HCV RNA levels according to ART and CTM in samples obtained 4 weeks after start of TVR/BOC therapy that yielded levels ≥50 IU/ml in at least one of the two assays.

		CTM	ART
TVR	1	2440	431
	2	2150	295
	3	665	237
	4	320	104
	5	190	35
	6	91	<12
	7	85	<12
	8	59	91
BOC	1	2620	38
	2	1570	36
	3	77	34
	4	70	<12

The discordant results in two samples (“TVR 1” and “TVR 2”) would have led to different treatment decisions (treatment discontinuation based on HCV RNA levels>1000 IU/ml at week 4). In a single sample the ART measured a higher HCV RNA level than the CTM (“TVR 8”).

At week 12 of TVR treatment only two out of three patients with an HCV RNA level above the threshold for treatment futility in the CTM test would have been withdrawn from treatment using the results from retesting with the ART (Table 3). Assay concordance was higher at week 8 after start of BOC treatment. Overall, 80% of the patients with an HCV RNA level>100 IU/ml in the CTM (n = 25) had HCV RNA levels above this limit also in the ART. However, this was mainly due to the fact that 40% had levels>1log above the recommended threshold for stopping of all medication (Table 4).

**Table 3:** HCV RNA levels according to ART and CTM in samples obtained 12 weeks after TVR treatment that yielded levels ≥50 IU/ml in at least one of the two assays.

		CTM	ART
TVR	1	17200	2502
	2	6990	2046
	3	4220	832
	4	959	186

The discordant results in one sample (“TVR 3”) would have led to a different treatment decision (treatment discontinuation based on HCV RNA levels>1000 IU/ml at week 4).

**Table 4:** HCV RNA levels according to ART and CTM measured in samples obtained 8 weeks after BOC treatment that yielded levels of ≥50 IU/ml in at least one of the two assays.

		CTM	ART
BOC	1	664000	371593
	2	176000	607115
	3	116000	44381
	4	28200	17099
	5	24300	32598
	6	7230	2556
	7	4660	2485
	8	1580	1027
	9	1120	430
	10	1080	229
	11	891	482
	12	840	356
	13	735	281
	14	510	217
	15	408	226
	16	397	132
	17	367	134
	18	344	177
	19	280	226
	20	151	105
	21	650	79
	22	529	67
	23	344	<12
	24	231	62
	25	176	59
	26	64	24
	27	52	31
	28	47	80

The discordant results in five samples (“BOC 21–25”) would have led to different treatment decisions (treatment discontinuation based on HCV RNA levels>100 IU/ml). In a single sample the ART measured a higher HCV RNA level than the CTM (“BOC 28”).

In contrast to weeks 4 and 12 of treatment, using the ART would have lead to more treatment discontinuations at week 24 than the CTM. With the CTM, 21 patients matched the recommended stopping criteria, which is any detectable HCV RNA at this stage. Retesting with the ART confirmed detectable HCV RNA in all of these samples except for a single one. In contrast, re-testing with the ART revealed detectable HCV RNA but below the LOQ (<12 IU/ml) in nine samples that were previously tested negative by CTM, as described earlier (Table 5). According to the ART, all nine patients should have stopped antiviral treatment based on current recommendations. Interestingly, SVR was achieved in four out of the eight patients (50%), for whom follow-up data were available. The majority of patients with detectable HCV RNA by CTM at week 24 were considered treatment failures according to the recommended stopping rules and withdrawn from treatment. In six patients, treatment was continued despite a detectable low viremic HCV RNA in the CTM. One patient was lost to follow up. The remaining five patients all experienced a relapse or breakthrough. Overall, sensitivity to identify a patient with a later treatment failure by residual viremia at week 24 was 33% using the ART and 20% with the CTM.

**Table 5:** Measured HCV RNA level by the ART and CTM in samples obtained 24 weeks after TVR/BOC therapy with a detectable HCV RNA in at least one of the two assays.

		CTM	ART
TVR	1	7290	2485
	2	3900	1073
	3	20	<12
	4	not detected	<12
	5	not detected	<12
	6	not detected	<12
BOC	1	3.150.000	1.341.040
	2	1.660.000	520.312
	3	73.700	36.725
	4	49.500	25.324
	*5*	45.100	49.652
	6	13.500	2.451
	7	12.800	8.845
	8	4.330	1.331
	9	2.100	599
	10	1.210	330
	11	687	87
	12	238	94
	13	<15	<12
	14	<15	<12
	15	<15	<12
	16	<15	<12
	17	<15	<12
	18	<15	not detected
	19	not detected	<12
	20	not detected	<12
	21	not detected	<12
	22	not detected	<12
	23	not detected	<12
	24	not detected	<12

The discordant results in ten samples (“TVR 4–6” and “BOC 18–24”) would have led to different treatment decisions (treatment discontinuation due to detectable HCV RNA). In a single sample the ART measured a higher HCV RNA level than the CTM (“BOC 5”).

## Discussion

On-treatment HCV RNA measurements are crucial for the prediction of a sustained virological response (SVR) and determination of treatment futility during the majority of modern HCV therapies. However, there are several commercially available HCV RNA assays, which differ in absolute HCV RNA quantification and lower detection limits [22], [23]. We here showed that there are significant differences in the performance of two HCV RNA tests, the Abbott RealTime test (ART) and the COBAS AmpliPrep/COBAS TaqMan HCV Test v2.0 (CTM) in classifying samples obtained from patients with advanced liver fibrosis or cirrhosis undergoing antiviral treatment with first generation HCV protease inhibitors (PIs). While both assays are comparable in their positive predictive value for SVR, there are remarkable differences in determining treatment futility. A significant number of successful viral clearances are missed if treatment is unnecessarily stopped due to detectable HCV RNA with the ART assay at week 24 of triple therapy, as it is currently recommended in the prescribing information and in international guidelines [21], while an almost equal number of patients were overtreated due to undetectable virus by CTM at this time point. In contrast, at earlier time points of PI-based triple therapy more patients may continue treatment due to lower absolute quantitative HCV RNA levels in the ART.

There are three aspects that are of particular importance for on-treatment HCV RNA measurement during most modern PI-based triple therapies: i) it is used to predict the likelihood for a later SVR, ii) to select patients for an abbreviated treatment regimen (response-guided therapy), and iii) to determine treatment futility. In the TVR and BOC registration trials, the chance of SVR was higher in patients with an undetectable HCV RNA result compared to those with detectable HCV RNA even at levels below the assays limit of quantification (LOQ) at several different time points of antiviral treatment [16]. However, in most DAA drug trials including the registration studies for BOC, TVR, as well as the second generation DAAs like simeprevir (SMV) and sofosbuvir (SOF), treatment response was measured with the Cobas TaqMan assay for use with the manual HighPure extraction kit (HPS). Contrarily, the HPS test is rarely used in routine clinical practice and seems to be slightly less sensitive in detecting low HCV RNA levels compared with the ART or the CTM [17], [24], [25]. We here confirmed in patients with advanced liver disease that an undetectable HCV RNA result early during therapy is a strong predictor for achieving SVR also if the CTM or the ART are used for HCV quantification. Of note, the ART was able to detect HCV RNA in several samples which produced an HCV RNA negative result in the CTM, while this was rarely observed the other way around. Similar results have been documented in a recently published study investigating the impact of the ART and CTM on response guided TVR-based triple therapy. Fewer patients would have been eligible for the shorter treatment duration if the ART would have been used to assess HCV RNA undetectability at week 4 due to a higher test sensitivity of this assay [26]. In our study, we focused on difficult-to-treat patients with advanced liver disease of whom in principle only a minority qualifies for shorter treatment duration. In these kinds of patients, it is crucial to ensure high SVR chances in order to justify the significant safety concerns that are associated with PI-based triple therapy. It was recently shown that patients with a repeatedly confirmed undetectable HCV RNA result during PI-based treatment have significantly higher chances to attain SVR compared with patients in whom repeated testing reveals detectable HCV RNA despite an initially undetectable HCV RNA result [17]. This was again observed in the present study. Patients with an undetectable HCV RNA result according to both ART and CTM had the highest chance for achieving SVR. However, despite the higher sensitivity of the ART and subsequently the larger number of samples identified as HCV RNA positive, the positive predictive value was not superior compared to the CTM. Overall, there was no clear advantage of using either of the assays in SVR prediction based on early HCV RNA measurements.

The second objective of our study addressed for the first time the impact of different performances of the ART and the CTM on stopping rules. Overall, there were two major findings. First, in samples with quantifiable HCV RNA, the ART tends to measure lower absolute HCV RNA levels. Consequently, more patients are likely to pass the HCV RNA cut-off of 1000 IU/ml at weeks 4 and 12 for TVR or 100 IU/ml at week 12 for BOC that are required to continue treatment. As the CTM was used to determine treatment futility in our patients, we were not able to study whether using the ART either leads to overtreatment of patients with poor chances for SVR or whether it prevents unnecessary early treatment discontinuations. However, in one patient who continued treatment despite formal futility at week 4 by CTM, but not by the ART assay, a breakthrough later during therapy was observed. Of course, no definite conclusion can be drawn form this observation in a single patient. To determine treatment futility it has to be considered that absolute cut-off levels for stopping rules have never been investigated in prospective trials. However, all patients with any quantifiable HCV RNA after more than four weeks of PI therapy had relatively low chances to achieve SVR during the pivotal trials [27]. In our study, all patients with quantifiable HCV RNA at week 8 or 12 experienced a treatment failure. This was the case even for those patients with levels below the recommended cut-offs in both assays. The second important finding was that, contrarily, at week 24 after start of PI therapy, residual HCV RNA was detected with the ART in several patients in whom the CTM produced HCV RNA negative results. All of these samples yielded levels below the LOQ of the ART (<12 IU/ml). Still, when referring to the current guidelines these patients would have been withdrawn from treatment according to the ART result [2], [20], [21]. However, half of these patients did ultimately achieve SVR. It has been suggested in other studies that at least at early time points of PI containing triple therapy a detectable HCV RNA result <12 IU/ml in the ART might be equal to an undetectable result with the CTM [26]. Based on our data, we believe that a detectable HCV RNA below the LOQ in the ART (<12 IU/ml detectable) at week 24 of TVR- or BOC-based triple therapy should not be considered as a necessary stopping rule. Instead, treatment continuation should be discussed in these patients based on their individual treatment associated risks. Whether residual viremia by the ART assay at later time points during treatment may be predictive for virological relapse has to be explored in future studies. It must be noted, however, that in a recent IFN-free DAA study residual HCV RNA could be detected by ART as late as end-of-treatment in some patients who subsequently achieved SVR [28].

Limitations of our study include the retrospective design and the relatively small patient number in certain subgroups. In addition, it has to be stated that all samples were first tested with the CTM and then retested with the ART. All patients were managed according to the respective HCV RNA result in the CTM. Thus, a direct comparison to patients that were treated according to the HCV RNA results by the ART was not possible. Due to limited sample volumes we were only able to test each sample once with the ART and the CTM. Thus, we were not able to study the impact of intra-assay variability. However, this question has been addressed in several other studies. In samples with quantifiable HCV RNA intra-assay variability were demonstrated to be low for both assays [29]–[31]. Per definition, a detectability rate of>95% is required at HCV RNA levels ≥LOD. In samples with levels below the LOD, intra-assay variability is significantly higher for both assays and varies depending on the respective HCV RNA level [17], [24], [29]–[31]. As BOC and TVR are only approved for HCV GT1, other HCV genotypes could not be studied here. We were also not able to analyze the impact of the HCV subgentoype (1a or 1b) as this information was not available for all patients an only a minority of the included patients was infected with HCV GT 1a.

Our data may be less relevant for some newer antiviral regimens including those containing the NS5B polymerase-inhibitor sofosbuvir (SOF) that have recently been approved in the US and some European countries, and which do not require on-treatment HCV RNA measurements [32], [33]. In countries with access to SOF GT1 patients can be treated with Peg-IFN/RBV/NUC triple therapy for a fixed treatment duration of 12 weeks. Due to the fact that the risk for viral resistance and a subsequent virological breakthrough is extremely low, there is no need for on-treatment HCV RNA measurements according to the prescribing information [32]. The same affects current and upcoming IFN free all oral DAA combination regimens. IFN free regimens consisting of SOF and the second generation PI Simeprevir (SMV) or a NS5A-inhibitor like daclatasvir (DCV) or ledipasvir (LDV) for a fixed, predetermined duration of 8 to 24 weeks have been shown to be highly effective also without the usage of RBV and even in patients with liver cirrhosis and a previous null response to Peg-IFN/RBV [34]–[38]. SMV is already available in several countries and the approval of DCV is expected soon. IFN free DAA combinations without SOF, i.e. including a NS5A-Inhibitor (ombitasvir), a non-nucleoside NS5B-Inhibitor (dasabuvir) and a PI (ABT-450 boosted with ritonavir) with or without RBV for 12 or 24 weeks also achieved high SVR rates of>90% [39]–[42]. Due to the very high efficacy and the excellent tolerability of these regimens, response-guided shortening or prolongation of therapy have not been studied and may not be needed to achieve high cure chances in the individual patient. However, given the high costs of direct antiviral drugs, HCV RNA testing during treatment may be helpful for surveillance of compliance and motivation of patients. Moreover, second generation DAAs including SOF are currently only available in very few countries due to the high costs and regulatory constraints. The vast majority of HCV patients currently have no access to these newer drugs and some are still awaiting the approval of TVR and BOC.

In summary we showed that both the Abbott RealTime Test and the COBAS AmpliPrep/COBAS TaqMan HCV Test v2.0 can be used to measure HCV RNA during antiviral treatment including telaprevir or boceprevir in order to predict the likelihood of a sustained virological response. However, differences in assay performances have to be considered. In particular the optimal HCV RNA cut-offs for the determination of treatment futility may differ depending on the used HCV RNA assay.

## Supporting Information

Table S1 All single HCV RNA test results and treatment outcome of the individual patients.
(PDF)
