# Supplementary material for: Performance of Two HCV RNA Assays during Protease Inhibitor-Based Triple Therapy in Patients with Advanced Liver Fibrosis and Cirrhosis
Source: PLoS One. 2014 Nov 12;9(11):e110857. doi: 10.1371/journal.pone.0110857 (PMC4229112; doi:10.1371/journal.pone.0110857)
Supplement: Table S1 — All single HCV RNA test results and treatment outcome of the individual patients. (PDF) [file pone.0110857.s001.pdf]

					HCV RNA result Week 4 of PI therapy (4 weeks after the end of the lead in)		HCV RNA result Week 12		HCV RNA result Week 24	
Patient ID	Protease Inhibitor	HCV GT	HCV subtype	Treatment Outcome	Abbott assay	Roche Asssay	Abbott assay	Roche Asssay	Abbott assay	Roche Asssay
P001	Telaprevir	1	b	Relapse	22	<15	<12	not detected	not detected	not detected
P002	Telaprevir	1	b	SVR	<12	not detected	not detected	not detected	not detected	not detected
P003	Telaprevir	1	b	Breakthrough	295	2150	2502	17200	NA	NA
P004	Telaprevir	1	b	SVR	<12	<15	not detected	not detected	not detected	not detected
P005	Telaprevir	1	a	SVR	not detected	not detected	not detected	not detected	not detected	not detected
P006	Telaprevir	1	b	SVR	not detected	not detected	not detected	not detected	not detected	not detected
P007	Telaprevir	1	a	Non responder	431	2440	NA	NA	NA	NA
P008	Telaprevir	1	b	Breakthrough	104	320	832	4220	NA	NA
P009	Boceprevir	1	b	Non responder	36	1570	226	280	NA	NA
P010	Boceprevir	1	b	SVR	<12	37	NA	NA	NA	NA
P011	Boceprevir	1	b	SVR	<12	<15	NA	NA	NA	NA
P012	Boceprevir	1	b	Non responder	NA	NA	NA	325	NA	NA
P013	Boceprevir	1	b	SVR	34	77	NA	NA	NA	NA
P014	Boceprevir	1	a	Breakthrough	<12	<15	<12	not detected	NA	NA
P015	Boceprevir	1	b	Non responder	<12	70	NA	NA	NA	NA
P016	Telaprevir	1	b	SVR	15	<15	not detected	not detected	not detected	not detected
P017	Telaprevir	1	b	SVR	not detected	not detected	not detected	not detected	not detected	not detected
P018	Telaprevir	1	b	SVR	12	<15	not detected	not detected	not detected	not detected
P019	Telaprevir	1	b	SVR	<12	not detected	not detected	not detected	<12	not detected
P020	Telaprevir	1	b	Breakthrough	not detected	not detected	not detected	not detected	NA	3338
P021	Telaprevir	1	b	Breakthrough	NA	NA	not detected	not detected	1073	3900
P022	Telaprevir	1	b	Relapse	<12	45	not detected	not detected	NA	NA
P023	Telaprevir	1	b	SVR	<12	<15	not detected	not detected	not detected	not detected
P024	Telaprevir	1	b	SVR	not detected	<15	not detected	not detected	not detected	not detected
P025	Telaprevir	1	b	SVR	14	<15	not detected	not detected	NA	NA
P026	Telaprevir	1	b	SVR	<12	<15	not detected	not detected	not detected	not detected
P027	Telaprevir	1	b	SVR	<12	not detected	not detected	not detected	not detected	not detected
P028	Telaprevir	1	NA	Breakthrough	35	190	not detected	<15	2485	7290
P029	Telaprevir	1	b	SVR	<12	<15	not detected	not detected	not detected	not detected
P030	Telaprevir	1	a	SVR	<12	not detected	not detected	not detected	not detected	not detected
P031	Boceprevir	1	b	Non responder	38	2620	79	650	NA	NA
P032	Boceprevir	1	b	SVR	<12	47	<12	<15	not detected	not detected
P033	Boceprevir	1	b	SVR	<12	<15	<12	<15	not detected	not detected
P034	Boceprevir	1	b	SVR	NA	NA	NA	NA	not detected	not detected
P035	Boceprevir	1	b	SVR	NA	NA	not detected	not detected	NA	NA
P036	Boceprevir	1	b	SVR	NA	NA	NA	not detected	not detected	not detected
P037	Telaprevir	1	b	SVR	<12	<15	not detected	not detected	not detected	not detected

P038	Telaprevir	1	b	SVR	<12	<15	not detected	not detected	not detected	not detected
P039	Telaprevir	1	a	Breakthrough	<12	not detected	not detected	not detected	<12	20
P040	Telaprevir	1	b	Relapse	not detected	<15	NA	not detected	<12	not detected
P041	Telaprevir	1	b	Relapse	not detected	not detected	NA	not detected	not detected	not detected
P042	Telaprevir	1	a	SVR	<12	not detected	not detected	not detected	not detected	not detected
P043	Telaprevir	1	b	Breakthrough	32	24	<12	*not detected*	NA	NA
P044	Telaprevir	1	b	Breakthrough	91	59	not detected	not detected	NA	NA
P045	Telaprevir	1	b	Breakthrough	not detected	<15	not detected	not detected	NA	NA
P046	Telaprevir	1	b	NA	not detected	not detected	not detected	not detected	NA	NA
P047	Telaprevir	1	b	SVR	<12	not detected	NA	not detected	not detected	not detected
P048	Telaprevir	1	a	Relapse	NA	NA	not detected	not detected	not detected	not detected
P049	Telaprevir	1	b	Non responder	237	665	NA	NA	NA	NA
P050	Telaprevir	1	b	SVR	not detected	not detected	not detected	not detected	not detected	not detected
P051	Telaprevir	1	b	SVR	not detected	not detected	not detected	not detected	not detected	not detected
P052	Telaprevir	1	b	SVR	<12	<15	not detected	not detected	not detected	not detected
P053	Telaprevir	1	b	SVR	17	<15	<12	not detected	not detected	not detected
P054	Telaprevir	1	b	SVR	not detected	not detected	not detected	not detected	not detected	not detected
P055	Telaprevir	1	a	Relapse	<12	<15	not detected	not detected	not detected	not detected
P056	Telaprevir	1	b	SVR	<12	<15	not detected	not detected	NA	NA
P057	Boceprevir	1	a	Non responder	146	390	97	170	NA	NA
P058	Boceprevir	1	b	SVR	55	160	NA	not detected	NA	NA
P059	Boceprevir	1	b	SVR	not detected	not detected	<12	not detected	NA	NA
P060	Boceprevir	1	b	SVR	20	<15	12	not detected	NA	NA
P061	Telaprevir	1	b	SVR	NA	<15	not detected	not detected	not detected	not detected
P062	Telaprevir	1	b	SVR	not detected	not detected	<12	not detected	<12	not detected
P063	Telaprevir	1	b	SVR	NA	not detected	not detected	not detected	not detected	not detected
P064	Telaprevir	1	a	Breakthrough	13	*33*	not detected	not detected	NA	NA
P065	Telaprevir	1	b	SVR	not detected	<15	<12	not detected	not detected	not detected
P066	Telaprevir	1	b	Relapse	<12	not detected	not detected	not detected	not detected	not detected
P067	Telaprevir	1	b	SVR	not detected	not detected	not detected	not detected	not detected	not detected
P068	Telaprevir	1	b	SVR	<12	not detected	not detected	not detected	not detected	not detected
P069	Boceprevir	1	a	Relapse	NA	NA	not detected	not detected	NA	NA
P070	Boceprevir	1	b	SVR	14	not detected	<12	not detected	NA	NA
P071	Boceprevir	1	a	SVR	not detected	not detected	not detected	not detected	NA	NA
P072	Boceprevir	1	b	Breakthrough	<12	<15	not detected	not detected	NA	NA
P073	Telaprevir	1	b	Non responder	<12	<15	not detected	*<15*	NA	NA
P074	Telaprevir	1	a	Breakthrough	<12	*91*	186	959	NA	NA
P075	Telaprevir	1	b	Breakthrough	<12	not detected	not detected	*not detected*	NA	NA
P076	Telaprevir	1	b	Breakthrough	<12	*85*	<12	*<15*	NA	NA
P077	Telaprevir	1	a	Relapse	<12	<15	not detected	not detected	not detected	not detected

P078	Telaprevir	1	b	SVR	not detected	not detected	not detected	not detected	not detected	not detected
P079	Telaprevir	1	b	Breakthrough	28	<15	not detected	not detected	NA	NA
P080	Telaprevir	1	a	SVR	<12	not detected	not detected	not detected	not detected	not detected
P081	Telaprevir	1	b	SVR	not detected	not detected	not detected	not detected	not detected	not detected
P082	Telaprevir	1	b	SVR	<12	<15	NA	not detected	NA	NA
P083	Telaprevir	1	b	SVR	<12	<15	NA	not detected	not detected	not detected
P084	Telaprevir	1	b	SVR	not detected	not detected	NA	not detected	not detected	not detected
P085	Telaprevir	1	b	Breakthrough	<12	<15	2046	6990	NA	NA
P086	Telaprevir	1	a	SVR	not detected	not detected	not detected	not detected	NA	NA
P087	Boceprevir	1	b	Breakthrough	NA	NA	62	231	2451	13500
P088	Boceprevir	1	b	Non responder	NA	NA	2556	7230	NA	NA
P089	Boceprevir	1	NA	SVR	NA	NA	not detected	not detected	not detected	not detected
P090	Boceprevir	1	b	SVR	NA	NA	NA	NA	not detected	not detected
P091	Boceprevir	1	a	Relapse	NA	NA	<12	not detected	not detected	not detected
P092	Boceprevir	1	NA	SVR	NA	NA	NA	NA	not detected	not detected
P093	Boceprevir	1	b	SVR	NA	NA	not detected	not detected	not detected	not detected
P094	Boceprevir	1	b	Breakthrough	NA	NA	59	176	36725	73700
P095	Boceprevir	1	b	SVR	NA	NA	NA	NA	not detected	not detected
P096	Boceprevir	1	b	Breakthrough	NA	NA	<12	22	<12	<15
P097	Boceprevir	1	NA	Breakthrough	NA	NA	<12	<15	<12	not detected
P098	Boceprevir	1	b	SVR	NA	NA	not detected	not detected	NA	NA
P099	Boceprevir	1	NA	SVR	NA	NA	not detected	not detected	NA	NA
P100	Boceprevir	1	a	Breakthrough	NA	NA	NA	NA	1331	4330
P101	Boceprevir	1	b	Relapse	NA	NA	NA	NA	not detected	not detected
P102	Boceprevir	1	b	Breakthrough	NA	NA	<12	not detected	330	1210
P103	Boceprevir	1	b	Relapse	NA	NA	not detected	not detected	not detected	not detected
P104	Boceprevir	1	b	SVR	NA	NA	not detected	not detected	not detected	not detected
P105	Boceprevir	1	b	SVR	NA	NA	not detected	<15	NA	NA
P106	Boceprevir	1	b	Relapse	NA	NA	NA	NA	not detected	not detected
P107	Boceprevir	1	NA	SVR	NA	NA	not detected	not detected	not detected	not detected
P108	Boceprevir	1	b	Relapse	NA	NA	not detected	not detected	not detected	not detected
P109	Boceprevir	1	b	SVR	NA	NA	not detected	not detected	not detected	not detected
P110	Boceprevir	1	NA	SVR	NA	NA	not detected	not detected	<12	not detected
P111	Boceprevir	1	b	NA	NA	NA	<12	<15	<12	not detected
P112	Boceprevir	1	a	SVR	NA	NA	not detected	not detected	not detected	not detected
P113	Boceprevir	1	NA	Breakthrough	NA	NA	not detected	not detected	NA	NA
P114	Boceprevir	1	b	Breakthrough	NA	NA	24	64	<12	<15
P115	Boceprevir	1	NA	Relapse	NA	NA	not detected	<15	<12	not detected
P116	Boceprevir	1	b	NA	NA	NA	80	47	not detected	not detected
P117	Boceprevir	1	b	Non responder	NA	NA	226	408	NA	NA

P118	Boceprevir	1	b	Non responder	NA	NA	217	510	NA	NA
P119	Boceprevir	1	a	Non responder	NA	NA	44381	116000	NA	NA
P120	Boceprevir	1	b	SVR	NA	NA	not detected	not detected	not detected	not detected
P121	Boceprevir	1	b	SVR	NA	NA	not detected	not detected	not detected	not detected
P122	Boceprevir	1	b	NA	NA	NA	not detected	not detected	not detected	not detected
P123	Boceprevir	1	b	Relapse	NA	NA	30	33	<12	not detected
P124	Boceprevir	1	b	Relapse	NA	NA	<12	<15	not detected	not detected
P125	Boceprevir	1	b	SVR	NA	NA	NA	NA	not detected	not detected
P126	Boceprevir	1	NA	Relapse	NA	NA	not detected	not detected	not detected	not detected
P127	Boceprevir	1	b	SVR	NA	NA	<12	<15	not detected	not detected
P128	Boceprevir	1	NA	SVR	NA	NA	<12	<15	not detected	not detected
P129	Boceprevir	1	b	Non responder	NA	NA	607115	176000	NA	NA
P130	Boceprevir	1	b	Relapse	NA	NA	NA	not detected	not detected	not detected
P131	Boceprevir	1	b	SVR	NA	NA	not detected	not detected	not detected	not detected
P132	Boceprevir	1	b	SVR	NA	NA	not detected	not detected	not detected	not detected
P133	Boceprevir	1	b	Relapse	NA	NA	<12	not detected	not detected	not detected
P134	Boceprevir	1	b	SVR	NA	NA	NA	NA	not detected	not detected
P135	Boceprevir	1	NA	Non responder	NA	NA	1027	1580	NA	NA
P136	Boceprevir	1	NA	Breakthrough	NA	NA	21	49	49652	45100
P137	Boceprevir	1	b	SVR	NA	NA	NA	NA	not detected	not detected
P138	Boceprevir	1	b	Non responder	NA	NA	177	344	NA	NA
P139	Boceprevir	1	b	Relapse	NA	NA	not detected	not detected	not detected	not detected
P140	Boceprevir	1	b	SVR	NA	NA	not detected	not detected	not detected	not detected
P141	Boceprevir	1	b	SVR	NA	NA	not detected	<15	not detected	not detected
P142	Boceprevir	1	b	Breakthrough	NA	NA	not detected	not detected	not detected	not detected
P143	Boceprevir	1	b	Non responder	NA	NA	229	1080	NA	NA
P144	Boceprevir	1	b	SVR	NA	NA	not detected	not detected	NA	NA
P145	Boceprevir	1	b	SVR	NA	NA	NA	NA	not detected	not detected
P146	Boceprevir	1	b	Non responder	NA	NA	67	529	NA	NA
P147	Boceprevir	1	b	SVR	NA	NA	not detected	not detected	not detected	not detected
P148	Boceprevir	1	NA	Non responder	NA	NA	2485	4660	NA	NA
P149	Boceprevir	1	b	Non responder	NA	NA	482	891	NA	NA
P150	Boceprevir	1	b	SVR	NA	NA	<12	not detected	not detected	not detected
P151	Boceprevir	1	b	Breakthrough	NA	NA	18	31	87	687
P152	Boceprevir	1	b	Non responder	NA	NA	32598	24300	NA	NA
P153	Boceprevir	1	b	Non responder	NA	NA	356	840	NA	NA
P154	Boceprevir	1	b	Relapse	NA	NA	NA	NA	not detected	not detected
P155	Boceprevir	1	NA	Non responder	NA	NA	371593	664000	NA	NA
P156	Boceprevir	1	NA	NA	NA	NA	not detected	<15	NA	NA
P157	Boceprevir	1	b	SVR	NA	NA	<12	<15	not detected	not detected

P158	Boceprevir	1	b	Breakthrough	NA	NA	31	52	94	238
P159	Boceprevir	1	b	Non responder	NA	NA	281	735	NA	NA
P160	Boceprevir	1	b	SVR	NA	NA	<12	<15	not detected	not detected
P161	Boceprevir	1	b	Breakthrough	NA	NA	<12	<15	599	2100
P162	Boceprevir	1	a	Non responder	NA	NA	17099	28200	NA	NA
P163	Boceprevir	1	b	SVR	NA	NA	NA	not detected	not detected	not detected
P164	Boceprevir	1	b	Non responder	NA	NA	<12	344	NA	NA
P165	Boceprevir	1	b	Breakthrough	NA	NA	not detected	20	NA	42900
P166	Boceprevir	1	NA	SVR	NA	NA	<12	<15	not detected	not detected
P167	Boceprevir	1	b	SVR	NA	NA	not detected	not detected	not detected	not detected
P168	Boceprevir	1	NA	Non responder	NA	NA	NA	NA	1341040	3150000
P169	Boceprevir	1	b	Breakthrough	NA	NA	not detected	not detected	520312	1660000
P170	Boceprevir	1	b	Non responder	NA	NA	430	1120	NA	NA
P171	Boceprevir	1	b	Breakthrough	NA	NA	<12	<15	8845	12800
P172	Boceprevir	1	b	SVR	NA	NA	NA	NA	<12	not detected
P173	Boceprevir	1	b	Relapse	NA	NA	NA	not detected	<12	<15
P174	Boceprevir	1	b	Breakthrough	NA	NA	NA	NA	<12	<15
P175	Boceprevir	1	b	SVR	NA	NA	not detected	not detected	not detected	not detected
P176	Boceprevir	1	b	Relapse	NA	NA	14	19	NA	not detected
P177	Boceprevir	1	NA	SVR	NA	NA	<12	not detected	not detected	not detected
P178	Boceprevir	1	b	NA	NA	NA	<12	<15	not detected	<15
P179	Boceprevir	1	b	SVR	NA	NA	NA	NA	not detected	not detected
P180	Boceprevir	1	a	SVR	NA	NA	not detected	not detected	not detected	not detected
P181	Boceprevir	1	NA	Relapse	NA	NA	<12	<15	not detected	not detected
P182	Boceprevir	1	NA	Breakthrough	NA	NA	<12	<15	25324	49500
P183	Boceprevir	1	b	SVR	NA	NA	NA	NA	not detected	not detected
P184	Boceprevir	1	b	Non responder	NA	NA	134	367	NA	NA
P185	Boceprevir	1	b	Relapse	NA	NA	not detected	<15	NA	NA
P186	Boceprevir	1	NA	SVR	NA	NA	not detected	not detected	not detected	not detected
P187	Boceprevir	1	b	Non responder	NA	NA	132	397	NA	NA
P188	Boceprevir	1	b	Non responder	NA	NA	105	151	NA	60
P189	Boceprevir	1	b	SVR	NA	NA	not detected	not detected	not detected	not detected
P190	Boceprevir	1	b	Relapse	NA	NA	<12	<15	<12	<15
P191	Boceprevir	1	b	Relapse	NA	NA	NA	NA	not detected	not detected

NA = not available
